# The physiological concentration of ferrous iron (II) alters the inhibitory effect of hydrogen peroxide on CD45, LAR and PTP1B phosphatases

**DOI:** 10.1007/s10534-015-9882-4

**Published:** 2015-09-25

**Authors:** Alicja Kuban-Jankowska, Magdalena Gorska, Lukasz Jaremko, Mariusz Jaremko, Jack A. Tuszynski, Michal Wozniak

**Affiliations:** Department of Medical Chemistry, Medical University of Gdansk, Gdansk, Poland; Max Planck Institute for Biophysical Chemistry and German Center for Neurodegenerative Diseases (DZNE), Göttingen, Germany; Department of NMR-based Structural Biology, Max Planck Institute for Biophysical Chemistry, Göttingen, Germany; Department of Physics, University of Alberta, Edmonton, Canada

**Keywords:** Ferrous iron, Hydrogen peroxide, CD45, LAR, PTP1B

## Abstract

Hydrogen peroxide is an important regulator of protein tyrosine phosphatase activity via reversible oxidation. However, the role of iron in this reaction has not been yet elucidated. Here we compare the influence of hydrogen peroxide and the ferrous iron (reagent for Fenton reaction) on the enzymatic activity of recombinant CD45, LAR, PTP1B phosphatases and cellular CD45 in Jurkat cells. The obtained results show that ferrous iron (II) is potent inhibitor of CD45, LAR and PTP1B, but the inhibitory effect is concentration dependent. We found that the higher concentrations of ferrous iron (II) increase the inactivation of CD45, LAR and PTP1B phosphatase caused by hydrogen peroxide, but the addition of the physiological concentration (500 nM) of ferrous iron (II) has even a slightly preventive effect on the phosphatase activity against hydrogen peroxide.

## Introduction

Protein tyrosine phosphatases (PTPs) are responsible for the regulation of tyrosine phosphorylation status controlling numerous cellular processes, such as cellular growth, differentiation, metabolism, cell–cell communication and immune response (Tonks 2006). One of the key representatives of PTPs is CD45, which controls many cellular processes and its overactivity is involved in autoimmune disorders, allergic response and carcinogenesis (Hermiston et al. 2009; Tan et al. 2000; Dios et al. 2005; Huntington and Tarlington 2004). CD45 is abundantly expressed in the Jurkat cell line (Zamoyska 2007). Phosphatases PTP1B is involved in pathogenesis of type 2 diabetes and obesity (Bence et al. 2006) and is a target in breast cancer treatment (Aceto and Bentires-Alj 2012). LAR phosphatase have also been implicated in metabolic regulation and cancer (Chagnon et al. 2004). CD45 and LAR are receptor-like PTPs predominantly found in the plasma membrane, while PTP1B is intracellular (cytosolic) phosphatase localized in a variety of intracellular compartments, such as cytosol, plasma membrane or endoplasmic reticulum (Andersen et al. 2001).

The hallmark defining the PTP superfamily is the strictly conserved active site sequence C(X)_5_R within the catalytic domain, which constitutes the phosphate-binding pocket of the enzyme (Tabernero et al. 2008). The cysteine residue inside the signature motif exists in the thiolate anion form, and is highly prone to oxidation (Pagliarini et al. 2004). Oxidation of the cysteine residue leads to the formation of a reversible form of sulfenic acid residue, while a highly oxidizing environment can induce further oxidation yielding physiologically irreversible sulfinic and sulfonic acid residues, all of which consequently cause inactivation of the enzyme (Ostman et al. 2011). Oxidative stress, defined as excessive reactive oxygen species (ROS) formation, may induce inactivation of protein tyrosine phosphatases. Inactivation via oxidation was suggested as a mechanism of protein tyrosine phosphatases regulation (Persson et al. 2004).

A unique biochemical and structural characteristic of the PTPs catalytic cysteine engendered a hypothesis that these enzymes might be direct targets of ROS chemistry. Many PTPs are shown to be oxidized transiently in response to various cellular stimuli. ROS such as hydrogen peroxide, function as second messengers and can regulate tyrosine phosphorylation-mediated signaling pathways (Finkel 2003).

**Fig. 1:**
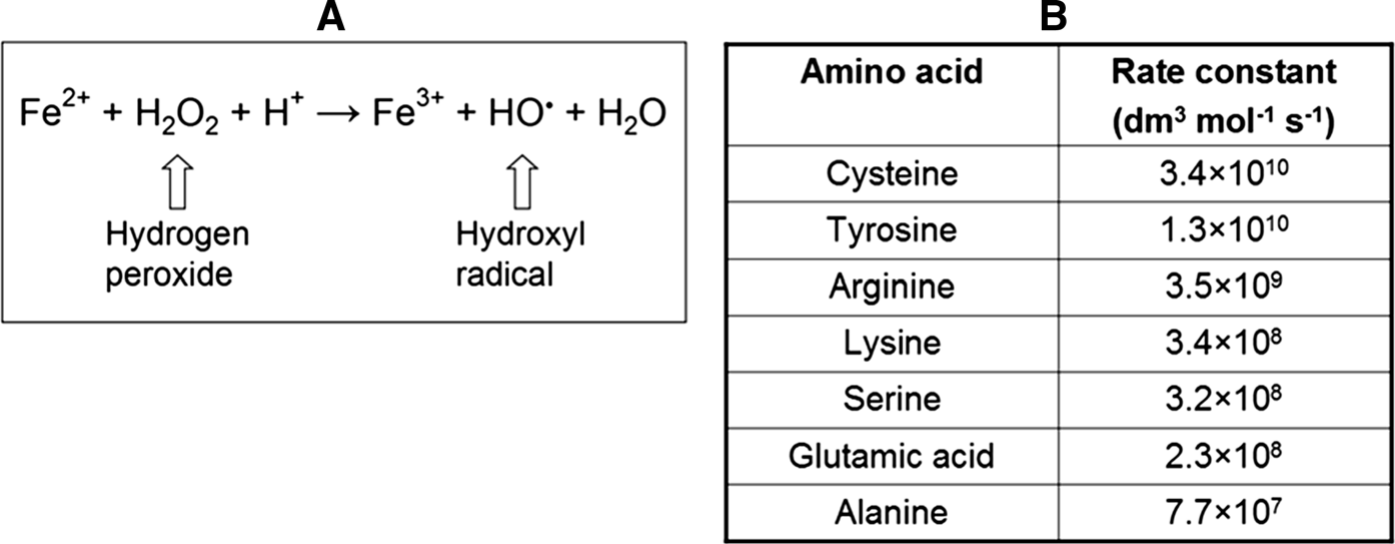
Hydrogen peroxide and ferrous iron in Fenton reaction chemistry. **a** Ferrous iron (II) is oxidized by hydrogen peroxide to ferric iron (III) and hydroxyl radical. **b** Rate constants for the reaction of hydroxyl radical with selected amino acids at pH 7 (Davies 2005)

Moreover, hydrogen peroxide may undergo spontaneous or enzymatic conversion to more potent oxidants (Klenk et al. 2000). We have recently identified that hydrogen peroxide, in the presence of carboxylic acids, efficiently activated to form peroxy acid, can induces inactivation of PTPs (Kuban-Jankowska et al. 2012, 2015; Bhattacharya et al. 2008). Due to the ubiquity of iron ions, hydrogen peroxide can lead to generation of highly reactive radicals such as hydroxyl radical during UV light exposure or by Fenton reaction in the presence of ferrous iron (II) (Winterbourn 2008). The Fenton reaction generally occurs in chemical and biological systems, but the nature of the oxidizing species obtained in a reaction observed by H.J.H. Fenton over one hundred years ago, is still not well understood (Barbusinski 2009). It was found as a key reaction in the oxidation of membrane lipids or amino acids. Importantly, the Fenton reaction is supposed to be involved in heart diseases, such as ischemia and reperfusion (Prousek 1995). In the Fenton reaction ferrous iron (II) is oxidized by hydrogen peroxide to ferric iron (III), a hydroxyl radical, and a hydroxyl anion (Fig. 1a). Hydrogen peroxide produced upon activation of many cell surface receptors is considered as a major regulator of PTPs in biological systems, but hydroxyl radical was also hypothesized to be physiologically relevant inhibitor of PTP1B phosphatase (Meng and Zhang 2013). Moreover, the intracellular iron after treatment with ROS may alters the signal transduction pathways (Deb et al. 2009).

Interestingly, the cysteine residue, which is essential for PTPs activity, is regarded as a main target for the hydroxyl radical. Comparison of the kinetic data for reactions of the hydroxyl radical with different amino acids (Fig. 1b), allows us an observation of the difference in oxidation. Although the variation in the rate constants is relatively small, the aromatic and sulfur-containing amino acids would be expected to be damaged more rapidly with the highest selectivity to cysteine residue (Davies 2005). Does selective diversity of the hydroxyl radical in biological systems (including PTPs) really exist? The high reactivity of hydroxyl radical is supposed to limit its ability to diffuse and cause active site—specific inactivation.

Hydrogen peroxide may relatively easily cross the cell membrane in response to insulin or epidermal growth factor, and control the cellular activity of protein tyrosine phosphatases therein (Rhee et al. 2000). Hydrogen peroxide is able to oxidize the catalytic cysteine residue to sulfenic acid, which can be reversibly reduced by various cellular reducing agents (Goldstein et al. 2005). The effect of hydrogen peroxide on activity of PTPs was already studied and described (Ross et al. 2007; Chiarugi and Cirri 2003). But the effect of the presence of ferrous iron (II), physiologically relevant reagent, was not been yet elucidated. The trace amounts of iron are presented in many cells. The potentially toxic labile iron exists in cells as a transit pool known as labile iron pool (LIP). It was estimated that the physiological concentration of LIP in Jurkat cells is around 3 ± 0.5 µM and that the treatment with hydrogen peroxide increased the cytosolic LIP levels (Al-Qenaei et al. 2014). Other studies showed that the estimated values of LIP for resting erythroid and myeloid cells are in the range of 0.2–1.5 µM (Epsztejn et al. 1997). Here we decided to compare the impact of hydrogen peroxide on recombinant phosphatases CD45, LAR, PTP1B and cellular CD45 from Jurkat cells in presence and in absence of ferrous iron at concentrations close to physiological.

## Materials and methods

### Cell line and cell culture

The human Jurkat T cell line, clone E.6-1, was obtained from European Collection of Cell Culture (ECACC, UK). The cells were cultured at 37 °C in RPMI 1640 medium supplemented with 10 % fetal bovine serum, 100 μg/mL penicillin/streptomycin and 2 mM l-glutamine. The culture was maintained at 37 °C and in an atmosphere containing 5 % CO_2_. RPMI 1640 medium and supplements were obtained from Sigma–Aldrich. The cell culture density was kept at 1 × 10^6^ cells/mL. At least every two days the medium was replaced with the fresh one, and the cells were counted and reseeded to maintain the recommended density. Concentration of protein in Jurkat cell lysate was measured using the Bradford colorimetric method. The Bradford method is based on Coomassie Brilliant Blue G-250 absorbance shift in the presence of protein. Binding to the protein being assayed under acidic conditions, the red dye is converted into the blue derivative. The amount of protein in the sample is proportional to the amount of bound dye, and thus to increase of an absorbance at 595 nm. Based on prepared standard concentrations of bovine serum albumin, concentration of protein in samples was calculated. Cells were suspended in FBS free RPMI 1640 medium before treatment with inhibitors.

### Cell viability assay with MTT

The Jurkat cells (1 × 10^6^ cells/mL) untreated (control) or treated with solution of hydrogen peroxide, FeSO_4_, or hydrogen peroxide together with FeSO_4_ (Fenton reaction) after the appropriate incubation time were suspended in solution of 0.5 mg/mL MTT(3-[4,5-dimethylthiazol-2-yl]-2,5 diphenyltetrazolium bromide) in RPMI 1640 without phenol red. The 100 µL samples were incubated for 2–4 h at 37 °C in 96-well plates. When the purple precipitate was clearly visible under the microscope, 100 µL of DMSO was added to each well and the plate with cover was left in the dark for 2–4 h. The absorbance at 540 nM was determined using a microplate reader.

### Determination of PTP CD45 activity in cell lysate

The Jurkat cells (density at 1 × 10^6^ cells/mL) were untreated (control) or treated with solution of hydrogen peroxide, FeSO_4_, or hydrogen peroxide together with FeSO_4_ (Fenton reaction) and incubated for 1 h at 37 °C in 24-well plates. The cells were rinsed twice with TBS, suspended at the density of 1 × 10^7^ cells/mL in Lysis buffer pH 7.4 with 0.5 % NP-40, 25 μg/mL leupeptin, 25 μg/mL pepstatin, 2 μg/mL aprotinin, 1 mM PMSF, vortexed briefly and placed on ice for 15 min. The cells were then solubilized by forcing the lysates through a 19-gauge needle (0.686 mm inner diameter) 20 times and centrifuged at 12000×*g* at 4 °C for 5 min. The supernatants were transferred to test tubes and assayed immediately. The day prior to the assay, the 96-well microplates were coated with CD45 capture antibodies (8 μg/mL in PBS) and incubated overnight at room temperature. After washing the wells, cell lysate was added, and the plate was placed on a rocking platform at 30 rpm for 3 h at room temperature. Lysates were aspirated from the wells and PTP activity was measured colorimetrically using 200 μM tyrosine phosphate specific substrate (phosphopeptide DADEY(PO3)LIPQQG in 10 mM HEPES buffer pH 7.4) and malachite green. The phosphopeptide substrate was dephosphorylated by active CD45 to generate unphosphorylated peptide and free phosphate. The free phosphate was then detected by a sensitive dye binding assay using malachite green and molybdic acid. The increase in absorbance at 620 nM was measured with the microplate reader. The activity of CD45 was determined by calculating the rate of phosphate release. CD45 capture antibody, tyrosine phosphate substrate DADEY(PO3)LIPQQG, malachite green and molybdic acid were purchased from R&D Systems. Detergent NP-40, protease inhibitors (leupeptin, pepstatin, aprotinin) and phenylmethylsulfonylfluoride (PMSF) were purchased from Sigma–Aldrich.

### Recombinant CD45, LAR and PTP1B activity assay

Human recombinant CD45 protein tyrosine phosphatase (PTP catalytic domain) was obtained from Sigma–Aldrich. Human LAR phosphatase (PTP catalytic domain) was obtained from Calbiochem. Human PTP1B phosphatase was purchased from Prospec. The solution of the recombinant protein tyrosine phosphatase CD45, LAR and PTP1B was prepared in 10 mM HEPES buffer pH 7.4. The final concentration of phospahatses in reaction samples was 0.8 μg/mL (10 nM). The CD45, LAR and PTP1B enzymes was untreated (control) or treated with solution of hydrogen peroxide, FeSO_4_, or hydrogen peroxide together with FeSO_4_ in different concentrations and) in the presence or absence of 1 mM EDTA. The assay was performed in 96-well microplates, and the final volume of each sample was 200 μL. The enzymatic activity of CD45, LAR and PTP1B was measured using 1 mM chromogenic substrate *para*-nitrophenyl phosphate (*p*NPP) in 10 mM HEPES buffer pH 7.4, at 37 °C. Phosphatase hydrolyzed *p*NPP to *para*-nitrophenol and inorganic phosphate. *Para*-nitrophenol is an intensely yellow colored soluble product under alkaline conditions. The increase in absorbance (due to *para*-nitrophenol formation) is linearly proportional to enzymatic activity concentration (with excessive substrate, i.e. zero-order kinetics) and was assessed at 405 nM on a microplate reader Jupiter (Biogenet) using DigiRead Communication Software (Asys Hitech GmbH).

### NBD-Cl modification assay

Sulfenic acid-labeling reagent 7-chloro-2-nitrobenzo-2-oxa-1,3-diazole (NBD-Cl) was purchased from Sigma. NDB-Cl react with both sulfenic acid and thiols forming adducts with different spectra. The amount of modified CD45, LAR, PTP1B thiol adduct with NBD (Cys-S-NBD adduct) was measured after 30 min incubation with NDB-Cl (0.6 mM in a 0.5 mL of sample) as absorbance in 347 and 420 nM with spectrophotometer.

### Statistical analysis

The experiments were performed at least three times. The data were applied and analyzed with GraphPad Prism (GraphPad Software v.4). Statistical analyses were performed using ANOVA combined with Tukey’s test, or *t* test. The data were expressed as mean ± SD. Differences between means were considered significant for P < 0.05.

## Results

To asses the effect of ferrous iron (II) and hydrogen peroxide we measured the enzymatic activity of recombinant CD45, LAR and PTP1B phosphatases under the cell-free conditions and CD45 phosphatase in Jurkat cells. The enzymes and cells were treated with solution of hydrogen peroxide, iron (II) sulfate, or both solutions together in different concentrations. Iron (II) sulfate (FeSO_4_) in aqueous solutions undergoes dissociation to ferrous iron (II) and sulfate ion (SO_4_^2−^).

### Comparison of the effect of hydrogen peroxide and ferrous iron on activity of recombinant CD45 phosphatase

In first step we decided to assess the effect of different concentrations of hydrogen peroxide on enzymatic activity of recombinant CD45 (data not shown) for calculation of IC_50_ value to plan the range of concentrations of hydrogen peroxide to be used in our studies. We calculated IC_50_ value for hydrogen peroxide as 8 µM, which is compatible with previous literature (Groen et al. 2005; Rider et al. 2003).

**Fig. 2:**
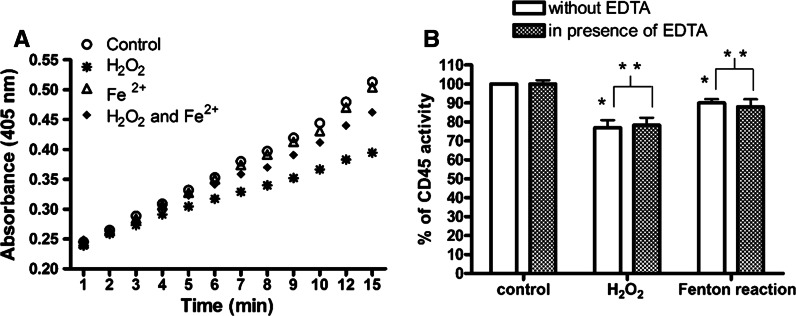
Recombinant CD45 inactivation mediated by hydrogen peroxide and ferrous iron. **a** CD45 activity after treatment with 5 µM hydrogen peroxide, 0.5 µM FeSO_4_ or hydrogen peroxide together with FeSO_4_ in presence of 1 mM pNPP. Data are presented as a mean ± SD (n = 3). One-way analysis of variance combined with Tukey test. **b** EDTA has no impact on enzymatic activity of recombinant CD45. The effect of presence of 1 mM EDTA on the activity of recombinant CD45 after 15 min incubation with 5 µM hydrogen peroxide, 0.5 µM FeSO4 or hydrogen peroxide together with FeSO4 (Fenton’s reagent). The results were presented as a percentage of control. Data presented as a means from three separate experiments. *Significantly different from control (P < 0.01) **Means were not significantly different in pairs (analyzed with t test, P > 0.05)

Then we compared the effect of hydrogen peroxide with ferrous iron (II) and we found that hydrogen peroxide induces inactivation of recombinant CD45 more effectively than in the presence of physiological concentration of ferrous iron (II). We observed that 5 µM hydrogen peroxide after 15 min of incubation inhibited 24 % of CD45 activity as compared to the control. The same concentration of hydrogen peroxide added together with 500 nM iron (II) sulfate decreased CD45 activity by 10 % (Fig. 2a). Incubation of recombinant phosphatase with solution of 500 nM iron (II) sulfate had virtually no effect on enzymatic activity (Fig. 1a). We tested the enzymatic activity of CD45 under the cell-free conditions in the presence and absence of 1 mM EDTA, but no statistically significant differences were observed between the activity of phosphatase treated with solution of hydrogen peroxide, iron (II) sulfate or Fenton’s reagent in the presence or absence of EDTA (Fig. 2b).

### Effect of ferrous iron on CD45 in Jurkat cells

**Fig. 3:**
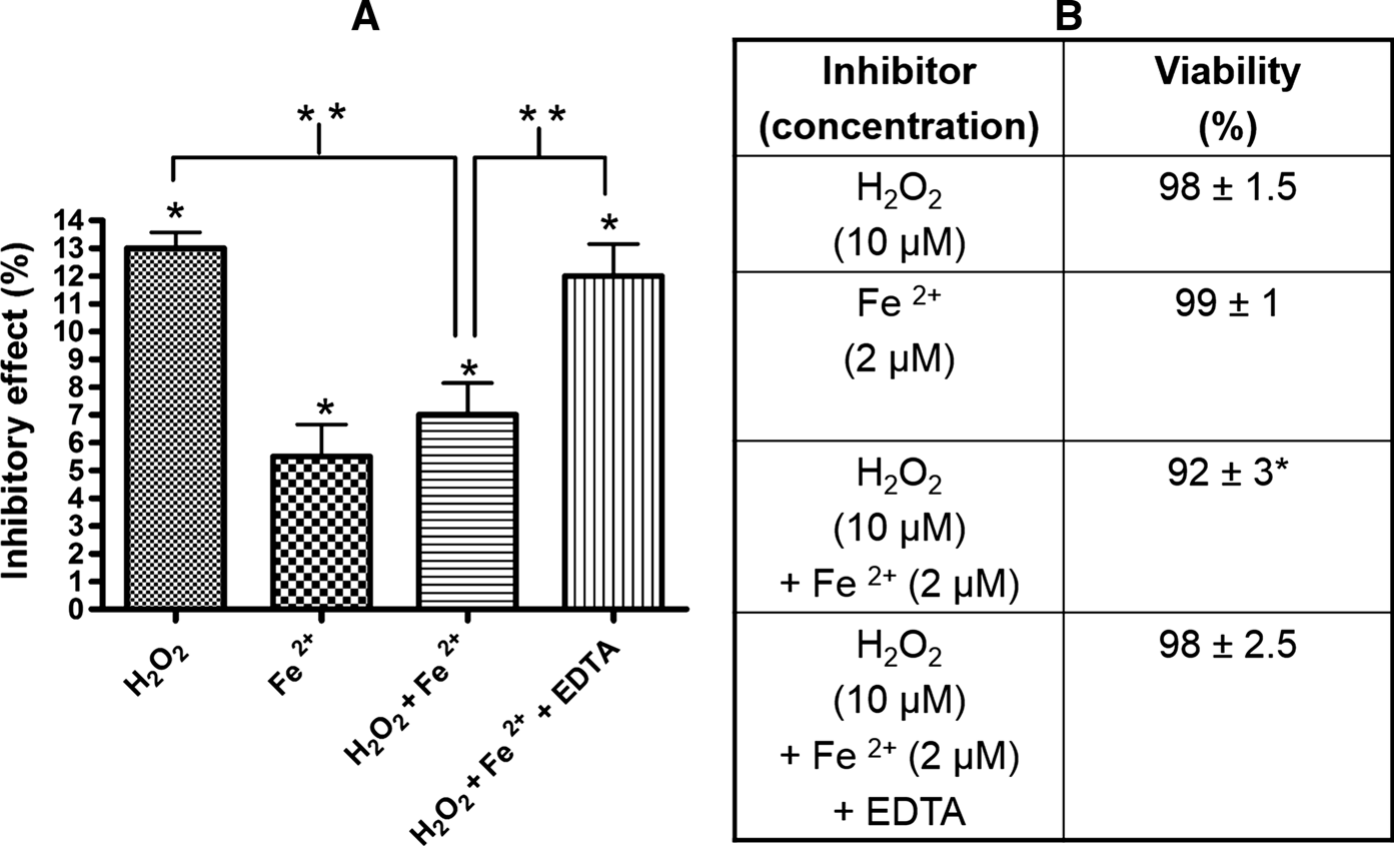
Impact of ferrous iron and hydrogen peroxide on the enzymatic activity of CD45 and cell viability in Jurkat cells. **a** The inhibitory effect of 10 µM hydrogen peroxide, 2 µM FeSO_4_ or hydrogen peroxide together with FeSO_4_ and with 1 mM EDTA on enzymatic activity of CD45 after treatment of Jurkat cells for 1 h. The results were presented as a percentage of inhibited activity comparing to control (as 0 %). Data presented as a mean ± SD (n = 3 independent experiments). One-way analysis of variance combined with Tukey test. *Significantly different (P < 0.001) from control. **Significantly different (P < 0.05). **b** Jurkat cells viability after treatment with 10 µM hydrogen peroxide, 2 µM FeSO_4_ or hydrogen peroxide together with FeSO_4_ or 1 mM EDTA for 1 h measured with MTT assay. The results were presented as a percentage of control. Data presented as a mean ± SD (n = 3). One-way analysis of variance combined with Tukey test. *Significantly different (P < 0.01) from control

The differences between the effect of hydrogen peroxide and the Fenton reaction were observed also for the CD45 activity assay in Jurkat cells. After 1 h of treatment of the cells with 10 µM hydrogen peroxide, 13 % of CD45 activity was inhibited, while incubation with the same concentration of hydrogen peroxide but in the presence of 2 µM iron (II) sulfate, decreased enzyme activity by 7 % (Fig. 3a). A solution of 2 µM iron (II) sulfate induces 5 % loss of CD45 activity in comparison to the control. Fenton’s reagent in the presence of EDTA more strongly reduced the enzymatic activity of CD45 than in the absence of EDTA (Fig. 3a). The effect on enzyme activity of Fenton’s reagent in the presence of EDTA (12 %) was similar to the effect of hydrogen peroxide (13 %).

The cell viability after 1 h of treatment of Jurkat cells with 10 µM hydrogen peroxide was not significantly changed (98 %), whereas the effect of the Fenton reaction on cell viability was slightly higher (92 %) (Fig. 3b).

### Effect of ferrous iron and hydrogen peroxide on recombinant phosphatase CD45, LAR and PTP1B

**Fig. 4:**
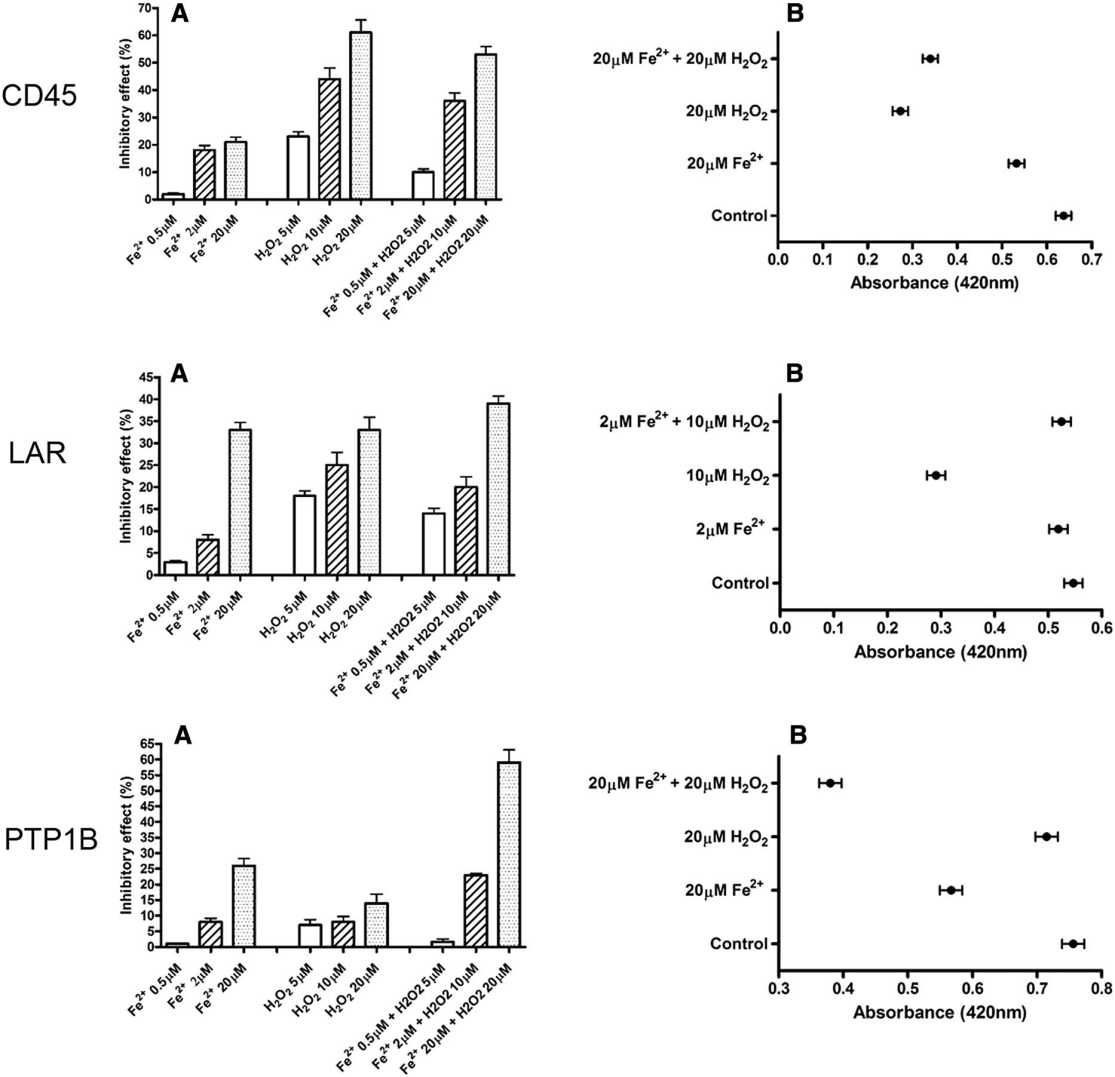
Impact of ferrous iron and hydrogen peroxide on the enzymatic activity of recombinant CD45, LAR, PTP1B phosphatases. **a** The inhibitory effect of 0.5, 2, 20 µM FeSO_4_, 5, 10, 20 µM hydrogen peroxide or hydrogen peroxide together with FeSO_4_ on recombinant CD45, LAR and PTP1B after 15 min of incubation. The results were presented as a percentage of inhibited activity comparing to control (as 0 %). Data presented as a mean ± SD (n = 3). **b** The amount of modified CD45, LAR, PTP1B thiol adduct with NBD (Cys-S-NBD adduct) as absorbance in 420 nm. One-way analysis of variance combined with Tukey test. *Significantly different (P < 0.05) from control. **Significantly different (P < 0.001)

We treated CD45, LAR and PTP1B recombinant phosphatase with different concentrations (0.5; 2; 20 µM) of iron sulfate combined with different concentrations of hydrogen peroxide (5; 10; 20 µM). The selected concentrations are presented in a Fig. 4a, b.

We demonstrated that both the hydrogen peroxide and ferrous iron (II) induce the inactivation of recombinant CD45, LAR and PTP1B and that the inhibitory effect was concentration dependent. Incubation of recombinant phosphatases with solution of 20 µM iron (II) sulfate significantly lowered the enzymatic activity, while 500 nM iron (II) sulfate had virtually no effect on enzymatic activity (Fig. 4a).

We observed also difference in the inactivation level between the phosphatases. PTP1B was considerably more sensitive for higher concentrations of iron (II) sulfate than hydrogen peroxide, while it was conversely for CD45 (Fig. 4a). We found that 20 µM iron (II) sulfate increased the effect of hydrogen peroxide on enzymatic activity of PTP1B. Interestingly, in our studies, we discovered that the low concentration (500 nM) of iron (II) sulfate decreases the inhibitory effect of hydrogen peroxide, paradoxically preventing PTPs from inactivation by hydrogen peroxide (Fig. 4a).

To examine the oxidation status of cysteine residues in studied PTPs we performed the thiol-labeling assay with NBD-Cl. We have measured the amount of NBD-Cl adducts with CD45, LAR and PTP1B thiol groups. As observed for CD45 and LAR phosphatase the amount of non-oxidized thiol groups was lower after treatment with hydrogen peroxide comparing to control and iron (II) sulfate and was increasing in addition of ferrous iron (Fig. 4b). The amount of non-oxidized thiol groups in PTP1B was lower after treatment with iron (II) sulfate than control and hydrogen peroxide. Moreover, the inhibitory effect of hydrogen peroxide in presence of iron (II) sulfate was significantly enhanced (Fig. 4b).

### Electrostatic potential of CD45, LAR and PTP1B active sites analysis

**Fig. 5:**
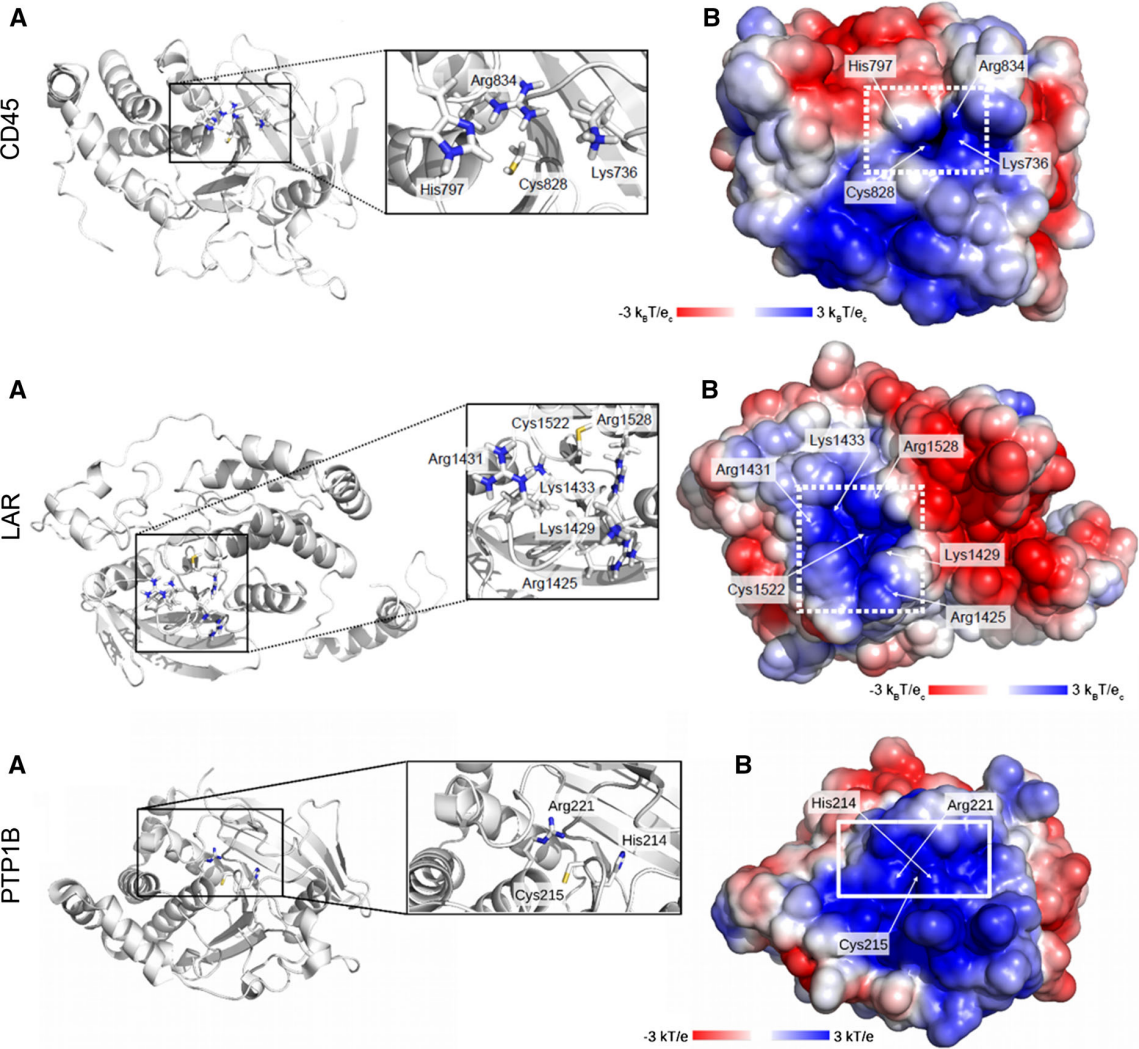
Positively charged catalytic pocket of tyrosine phosphatase CD45, LAR, PTP1B. **a** The ribbon representation of the D1 domain from tyrosine phosphatase CD45, LAR and PTP1B. **b** The Van der Waals surface of D1 domain from the CD45, LAR, PTP1B receptor protein colored according to the surface potential, calculated using APBS (Baker et al. 2001). *Blue* indicates positive while *red* negative charge. Catalytic Cys828, His797 and two neighboring positively charged residues Lys736 and Arg834 are marked in sticks. The same protein orientations are in** a** and** b**, in** b** white dotted square corresponds to the *black square* in** a** and indicates the entrance to the catalytic center. Protein coordinates for CD45 were taken from PDB 2YGU from Glu599 to Gly891. All seleno-methionines were replaced by methionines and Ser828 was replaced by cysteine. Protein coordinates for LAR were taken from PDB 1LAR and for PTP1B from PDB 1SUG. (Color figure online)

In order to determine if the ferrous iron is able to penetrate into the active sites of CD45, LAR and PTP1B we decided to analyze the electrostatic surface of the phosphatase catalytic domains. The computational study showed that the surface of the active site of CD45 is highly electropositive and there are amino acids residues (Arg834, His797, Lys736) with positive charge limiting access to the catalytic cysteine (Fig. 5a). This suggests that the electrostatic repulsion partially prevents ferrous iron (II) from entering into the catalytic center of CD45, whereas hydrogen peroxide may easily penetrate into the catalytic cysteine inducing its oxidation.

The similar electrostatic potential calculations were performed for the active sites of LAR and PTP1B. We showed that the surface of LAR and PTP1B active sites are largely positively charged and there are only slight differences found between surfaces of active site surrounding (Fig. 5b, c).

## Discussion

Here we showed that ferrous iron (II) can induce inactivation of recombinant CD45, LAR, PTP1B and cellular CD45 phosphatase in Jurkat cells. We found that the effect of ferrous iron (II) was concentration dependent.

We demonstrate that the physiological concentrations of ferrous iron (II) has lower inhibitory effect on CD45, LAR and PTP1B phosphatase than hydrogen peroxide. Moreover, in our studies, the addition of low concentration of ferrous iron to hydrogen peroxide paradoxically exhibits a slightly preventive effect.

**Fig. 6:**
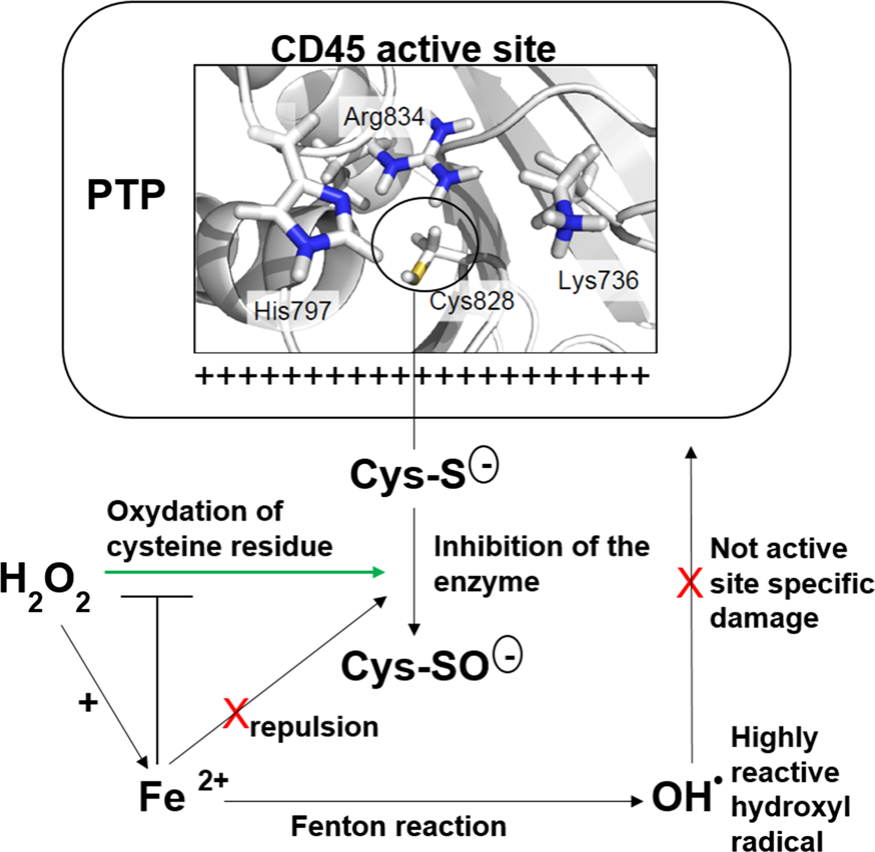
Schematic representation of the proposed mechanism of hydrogen peroxide inactivation of PTP CD45, possible electrostatic repulsion of ferrous iron (II) and unspecific damage caused by hydroxyl radical formed in Fenton reaction

We suggest that stronger effect of low concentrations of hydrogen peroxide is due to the fact it may easily penetrate to PTP active site inducing inactivation of enzymatic activity, while small amounts of ferrous iron are not able to reach the PTPs active site (Fig. 6). Computational analysis of the CD45, LAR and PTP1B active sites allowed us to assume that the electropositive surface of the active site residues can cause repulsion of positively charged ferrous iron (Fig. 5). Presumably, higher concentrations of ferrous iron allow it to place closer to the active site of PTPs and induce inactivation. Preventive effect of low concentrations of ferrous iron on the phosphatase activity against hydrogen peroxide may be explained due to the removal of hydrogen peroxide from the reaction environment by absorbing hydrogen peroxide into Fenton reaction (Fig. 6). Highly reactive hydroxyl radical, formed in Fenton reaction, is only able to cause unspecific damage and will not reach the catalytic cysteine in the active site.

The weaker effect of low concentrations of ferrous iron (II) than hydrogen peroxide was observed also for CD45 phosphatase in Jurkat cells (Fig. 3a). We suggest that ferrous iron (II) due to electrostatic potential of PTPs active site, is not able to reach the intracellular target, while hydrogen peroxide would easily cross the cell membrane of Jurkat cells and penetrate to PTP active site inducing inactivation of enzymatic activity. The fact that the impact of ferrous iron with hydrogen peroxide in the presence of EDTA in Jurkat cells was similar to the effect of hydrogen peroxide confirmed the paradoxically protective role of the low amounts of ferrous iron on the activity of phosphatase CD45. EDTA has virtually no impact on the enzymatic activity of recombinant CD45 (Fig. 2b), but enhances the inhibitory effect of hydrogen peroxide in presence of ferrous iron in Jurkat cells, probably by chelating the extracellular ferrous iron derived from the cell treatment and allowing free penetration by hydrogen peroxide. The other explanation may be the formation of iron chelate complex (FeEDTA) leading to the initiation of other strong oxidants or lipid peroxidation (Bucher et al. 1983a, b) and consequently to the oxidation of CD45 phosphatase (Conrad et al. 2010).

We found that addition of ferrous iron (II) to hydrogen peroxide has slightly decreases the viability of Jurkat cells (Fig. 3b). Further studies are required to investigate this problem. The hydroxyl radical form from hydrogen peroxide in presence of ferrous iron is highly reactive and may react unspecifically with all structures of cells inducing damage of DNA and proteins. It has previously been noted that the viability of Jurkat cells can be decreased by oxidative DNA damage in cells caused by hydroxyl radicals generated in the Fenton reaction (Riviere et al. 2006; Tenopoulou et al. 2005).

The comparison of the effect of ferrous iron and hydrogen peroxide on activity of recombinant CD45, LAR and PTP1B showed that the impact of ferrous was concentration and phosphatase dependent. The addition of higher concentrations of ferrous iron to hydrogen peroxide led to increase of inactivation, especially of PTP1B phosphatase.

**Fig. 7:**
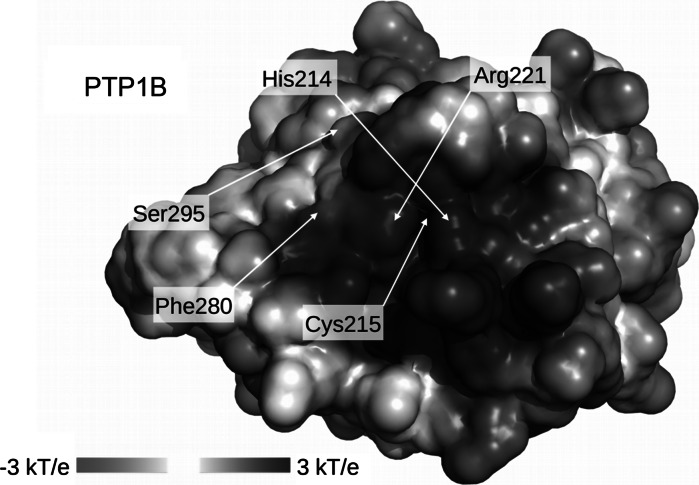
The allosteric binding site of PTP1B represented by Phe250 and Ser295 residues and the active site with catalytic Cys215, Arg221 and His214. The Van der Waals surface of D1 domain of PTP1B colored according to the surface potential, calculated using APBS (Baker et al. 2001). *Blue* indicates positive and *red* negative charge. Protein coordinates for PTP1B from PDB 1SUG. (Color figure online)

Although the electrostatic potential on a surface of active sites is similar for CD45, LAR and PTP1B and largely positively charged, there are many differences in a structural surface of those phosphatases (Barr et al. 2009). The diverse regions include the topology of second binding site, contributing to substrate specificity and may explain slight differences in sensitivity of selected phosphatases. The higher vulnerability of PTP1B may be due to possible allosteric regulation of the activity of PTP1B. It was found that small-molecule inhibitors may bind in a allosteric binding site of PTP1B located 20 A˚ from the active site (Tabernero et al. 2008) and do not have negative charges (Zhang and Zhang 2007). Allosteric inhibition blocks the mobility of catalytic WPD-loop and stabilize the inactive conformation of PTP1B (Wiesmann et al. 2004; Hansan et al. 2005). Previous crystallographic studies have shown that PTP1B can exist in two conformations. In the native form, the WPD-loop is in an “open” conformation, and the binding pocket is easily accessible to substrate. Upon substrate binding, the WPD-loop closes over the active site, forming a tight binding pocket for the substrate (Barford et al. 1994; Jia et al. 1995). The WPD-loop closure is essential for the catalytic mechanism of PTP1B (Kolmodin and Aqvist 2001). Unlike catalytic site, the allosteric site is not well conserved and substantially less polar. The allosteric binding site of PTP1B possessing neutral surface is presented in Fig. 7, with Phe250 and Ser295 residues. The presence of secondary binding pocket can be one of possible explanation for the small differences in inhibitory level.

In conclusion, hydrogen peroxide produced upon activation of many cell surface receptors is considered as a major regulator of PTPs in biological systems. Here, we demonstrate that ferrous iron (II) may be considered as inhibitor of PTPs. In this studies, we present how different concentration of iron may alter inhibitory effect of hydrogen peroxide. We also propose that probably electrostatic potential of proteins surface protects enzymes from the chemistry of elements present in the surrounding environment.
